# Integrated Proteome and Phosphoproteome Profiling Reveals Abundance-Independent Phosphorylation Remodeling in Hepatocellular Carcinoma

**DOI:** 10.3390/ijms27208977

**Published:** 2026-10-10

**Authors:** Shafaq Saleem, Muhammad Salman Sajid, Rency S. Varghese, Lin Liu, Vinona Muralidaran, Alexander Kroemer, Habtom W. Ressom

**Affiliations:** 1 Department of Oncology, Lombardi Comprehensive Cancer Center, Georgetown University Medical Center, Washington, DC 20057, USA; 2 MedStar Georgetown Transplant Institute, MedStar Georgetown University Hospital, Washington, DC 20057, USA; 3 The Center for Translational Transplant Medicine, Georgetown University Medical Center, Washington, DC 20057, USA

**Keywords:** proteomics, phosphoproteomics, biomarker, human tissue, nano-LC-MS/MS

## Abstract

Hepatocellular carcinoma (HCC) is characterized by extensive molecular heterogeneity, yet changes in protein abundance alone may not fully capture the regulatory alterations that drive tumor biology. Here, we performed integrated whole-proteome and phosphoproteome profiling of paired HCC and adjacent non-tumorous liver tissues to distinguish protein-abundance remodeling from phosphorylation changes not explained by comparable parent-protein abundance changes. Label-free nano-LC-MS/MS data were processed using quality control, filtering, missing-value imputation, normalization, and paired statistical testing. Whole-proteome analysis retained 3653 proteins and identified 314 significantly altered proteins, including 79 increased and 235 decreased proteins in HCC. Phosphoproteome analysis retained 1682 phosphopeptides and identified 189 significantly altered phosphopeptides, including 81 increased and 108 decreased events. Integrated analysis showed that 132 significant phosphopeptides matched quantified parent proteins; 112 were classified as phospho-only events, and 20 showed concordant phosphopeptide and parent-protein changes. Reactome enrichment of phospho-only parent proteins highlighted immune/inflammatory processes, metabolic remodeling, ER-stress/protein-folding responses, apoptosis, RHO/RHOBTB GTPase-related regulation, and post-translational phosphorylation. Candidate prioritization identified phosphorylation events for future validation. Overall, this paired multi-layer proteomic analysis demonstrates that HCC tissue exhibits both protein-abundance remodeling and phosphorylation-level rewiring, supporting integrated proteome–phosphoproteome profiling as a strategy to resolve regulatory alterations in cancer tissue.

## 1. Introduction

Hepatocellular carcinoma (HCC) is the predominant form of primary liver cancer and remains a major cause of cancer-related mortality worldwide. Its clinical behavior is shaped by substantial molecular heterogeneity, which complicates biological interpretation, limits robust patient stratification, and makes it difficult to define signaling programs that consistently drive tumor progression [1,2]. Although genomic and transcriptomic studies have substantially advanced the current understanding of HCC, these layers do not fully capture the functional state of tumor cells [3,4]. The relationship between nucleic acid abundance and protein function is influenced by translational control, protein turnover, post-translational modification, and pathway-dependent regulation, all of which can reshape tumor biology downstream of gene expression.

Proteomic analysis therefore provides a more direct view of the molecular programs that shape tumor behavior, including pathway activity, metabolic remodeling, stress adaptation, and microenvironment-associated processes [5]. Recent integrative studies have shown that clinically relevant biology can be resolved more clearly at the proteomic level than at the nucleic acid level alone [3,4]. Among proteomic layers, the phosphoproteome is particularly informative because phosphorylation is a central regulator of signal transduction, protein activity, localization, interaction dynamics, and cellular adaptation to environmental and oncogenic stress. In cancer, many biologically important processes, including proliferation, survival signaling, growth factor response, cell-cycle control, and metabolic reprogramming, are mediated through phosphorylation-dependent mechanisms [6,7].

Recent advances in mass spectrometry-based phosphoproteomics have improved sensitivity, depth, and quantitative coverage, making it increasingly practical to interrogate pathway-level signaling states in complex cancer samples. Modern phosphoproteomic workflows now enable increasingly deep and biologically interpretable measurements of signaling regulation and kinase-driven pathway activity in cancer systems [8]. This issue is especially important in HCC, where tumor progression is closely linked to dysregulated growth-factor signaling, kinase activity, and metabolic rewiring [3,4]. Integrated proteogenomic analysis of HCC across etiologies and stages has shown that signaling, metabolism, and microenvironmental programs can diverge substantially across molecular layers, and that the phosphoproteome adds information that is not captured by protein abundance alone [3]. These observations are highly relevant in HCC because many pathways implicated in tumor growth and progression, including receptor tyrosine kinase signaling, PI3K-AKT-mTOR signaling, MAPK signaling, and broader metabolic regulatory networks, are strongly modulated through phosphorylation-dependent control [4].

However, phosphoproteomic signals are often interpreted without sufficiently considering the abundance of the corresponding proteins. An increase in phosphopeptide signal may represent a true regulatory phosphorylation event, but it may also result simply from increased abundance of the parent protein [9,10]. Likewise, a decrease in phosphopeptide abundance may reflect either loss of phosphorylation or reduced abundance of the protein carrying that site. Conversely, phosphorylation changes that occur in the absence of matched protein-level changes may reflect abundance-independent phosphoregulation and therefore provide stronger evidence of signaling rewiring [11]. Integrated analysis of whole-proteome and phosphoproteome data therefore provides a more rigorous framework for distinguishing abundance-dependent phosphorylation from abundance-independent regulatory events and for identifying concordant and discordant patterns across molecular layers [3,9,10,11].

In this study, we performed integrated whole-proteome and phosphoproteome profiling of paired HCC and non-tumorous liver tissues to define both protein-abundance remodeling and phosphorylation-level regulation associated with HCC. By mapping significantly altered phosphopeptides to quantified parent proteins, we distinguished phosphorylation changes that could be interpreted in the context of protein abundance from those occurring independently of measurable parent protein regulation. This strategy revealed a prominent abundance-independent phosphorylation signature in HCC, highlighted dysregulated oncogenic signaling pathways, and enabled prioritization of phospho-only candidate events for future validation.

## 2. Results

**Figure 1:**
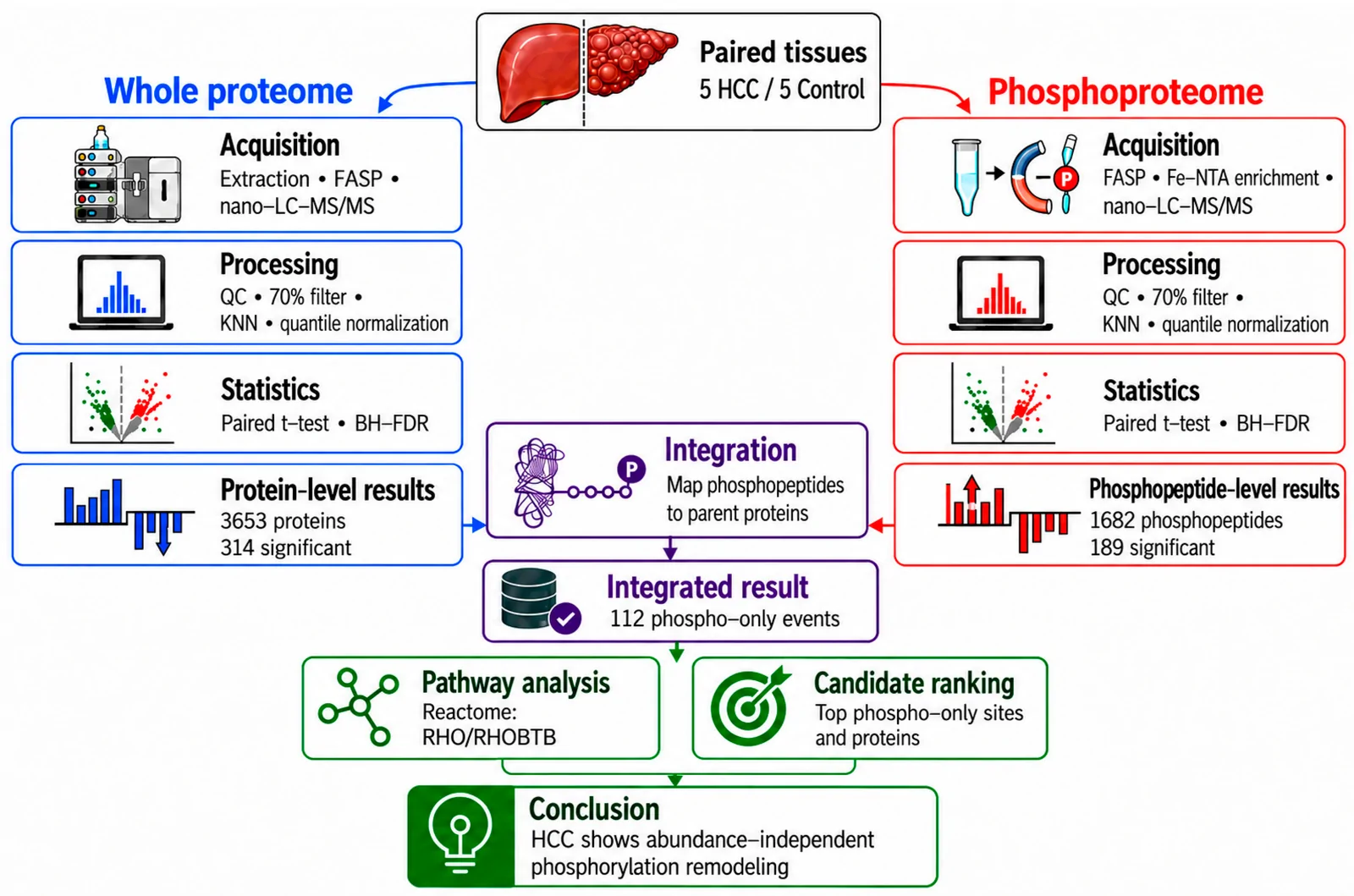
Integrated proteome–phosphoproteome workflow for paired HCC tissue analysis.

To define protein-abundance and phosphorylation-level alterations associated with HCC, we performed an integrated whole-proteome and phosphoproteome analysis of paired HCC and adjacent non-tumorous tissue samples. The overall experimental and computational workflow is summarized in Figure 1. Briefly, paired tissue specimens were analyzed by label-free nano-LC-MS/MS to generate protein-level and phosphopeptide-level abundance datasets. Each data layer was independently processed using quality-control assessment, filtering, missing-value imputation, normalization, and paired statistical testing to account for the matched study design. Technical-replicate quality control, detection-count evaluation, correlation analysis, and normalization comparison supported the reproducibility and suitability of both datasets for downstream analysis. Differentially regulated phosphopeptides were then mapped to their corresponding parent proteins quantified in the whole proteome dataset to distinguish phosphorylation changes associated with altered protein abundance from abundance-independent phosphorylation events.

### 2.1. Whole-Proteome Profiling Identifies Protein-Abundance Remodeling in HCC Tissue

We first analyzed the whole-proteome dataset (Table S1) to define global protein-abundance alterations in HCC vs. adjacent tissues. Technical replicate quality control showed consistent quantitative performance across LC-MS/MS runs, supporting replicate-level summarization before biological sample-level analysis (Figures S1 and S2). Detection-count assessment further demonstrated consistent protein recovery across technical injections and paired tissue samples (Figure S3). Correlation heatmaps showed strong similarity among technical replicates, indicating that technical variability was not the primary driver of the observed biological differences (Figure S4). Normalization comparison was then used to assess sample-level intensity distributions and reduce systematic quantitative differences before paired statistical testing (Figure S5).

**Figure 2:**
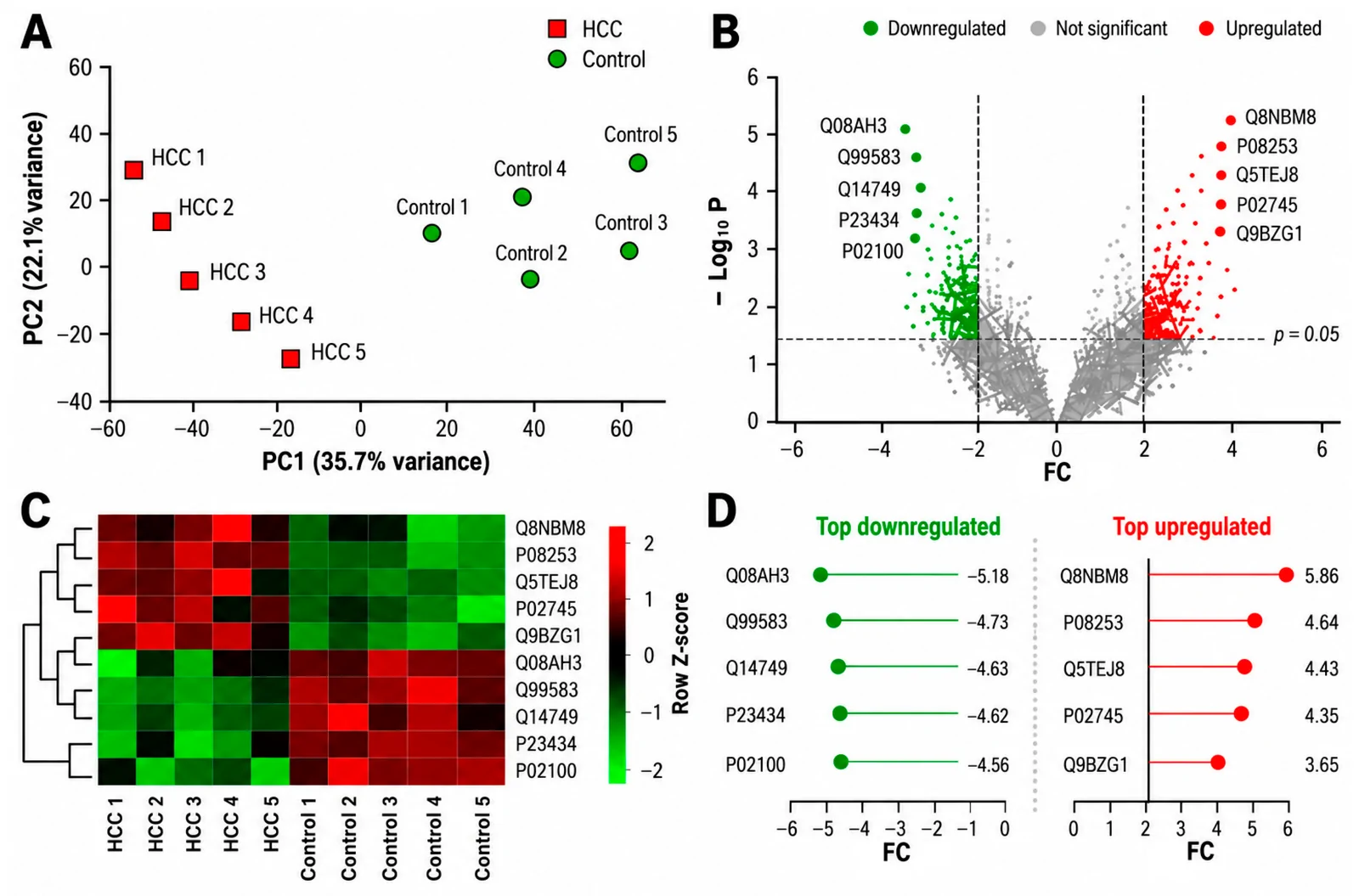
Whole-proteome remodeling in HCC tissue. (**A**) PCA of HCC and control samples. (**B**) Volcano plot of altered proteins (significantly increased and decreased proteins are in red and green, respectively). (**C**) Heatmap of significant proteins. (**D**) Top 5 upregulated and downregulated proteins in HCC. FC is defined as the log_2_-transformed ratio of HCC to control values.

After filtering, 3653 proteins were retained, on which we applied missing-value imputation and normalization prior to using a paired t-test for differential-abundance analysis. Principal component analysis (PCA) showed separation between HCC and adjacent non-tumorous samples, indicating that the whole-proteome abundance profiles captured disease-associated molecular differences between paired tissue groups (Figure 2A). Differential-abundance analysis identified 976 proteins altered at a nominal significance threshold of *p* < 0.05. We identified 314 proteins significantly altered in HCC compared with adjacent normal tissues (false discovery rate [FDR] < 0.10 and |FC| ≥ 2, where FC is defined as the log_2_-transformed ratio of the HCC to control abundance values). These include 79 proteins that increased and 235 proteins that decreased relative to adjacent non-tumorous tissue (Figure 2B,D) detailed in Table S2.

The volcano plot in Figure 2B demonstrates broad remodeling of the HCC tissue proteome. Hierarchical clustering of the 314 significant proteins separates HCC and control samples into distinct abundance patterns, supporting the biological relevance of the identified protein-abundance changes (Figure 2C). The ranked display of the most altered proteins further highlights proteins with the largest positive and negative FC values (Figure 2D). Together, these findings show that HCC tissue exhibits measurable whole-proteome remodeling and provide a protein-abundance reference layer for phosphoproteome integration.

### 2.2. Phosphoproteome Profiling Reveals Extensive Phosphorylation Remodeling in HCC Tissue

We next analyzed the phosphopeptide-level dataset (Table S3) to determine whether HCC tissue also exhibits phosphorylation-level remodeling. Similar to the whole-proteome workflow, quality control of technical replicates supported strong reproducibility across phosphoproteome LC-MS/MS runs (Figures S6 and S7). Detection-count plots showed reproducible phosphopeptide recovery following enrichment (Figure S8), and technical-run correlation heatmaps supported consistent phosphopeptide quantification across replicate injections (Figure S9). Normalization assessment confirmed that phosphopeptide abundance distributions were suitable for downstream paired statistical analysis (Figure S10).

**Figure 3:**
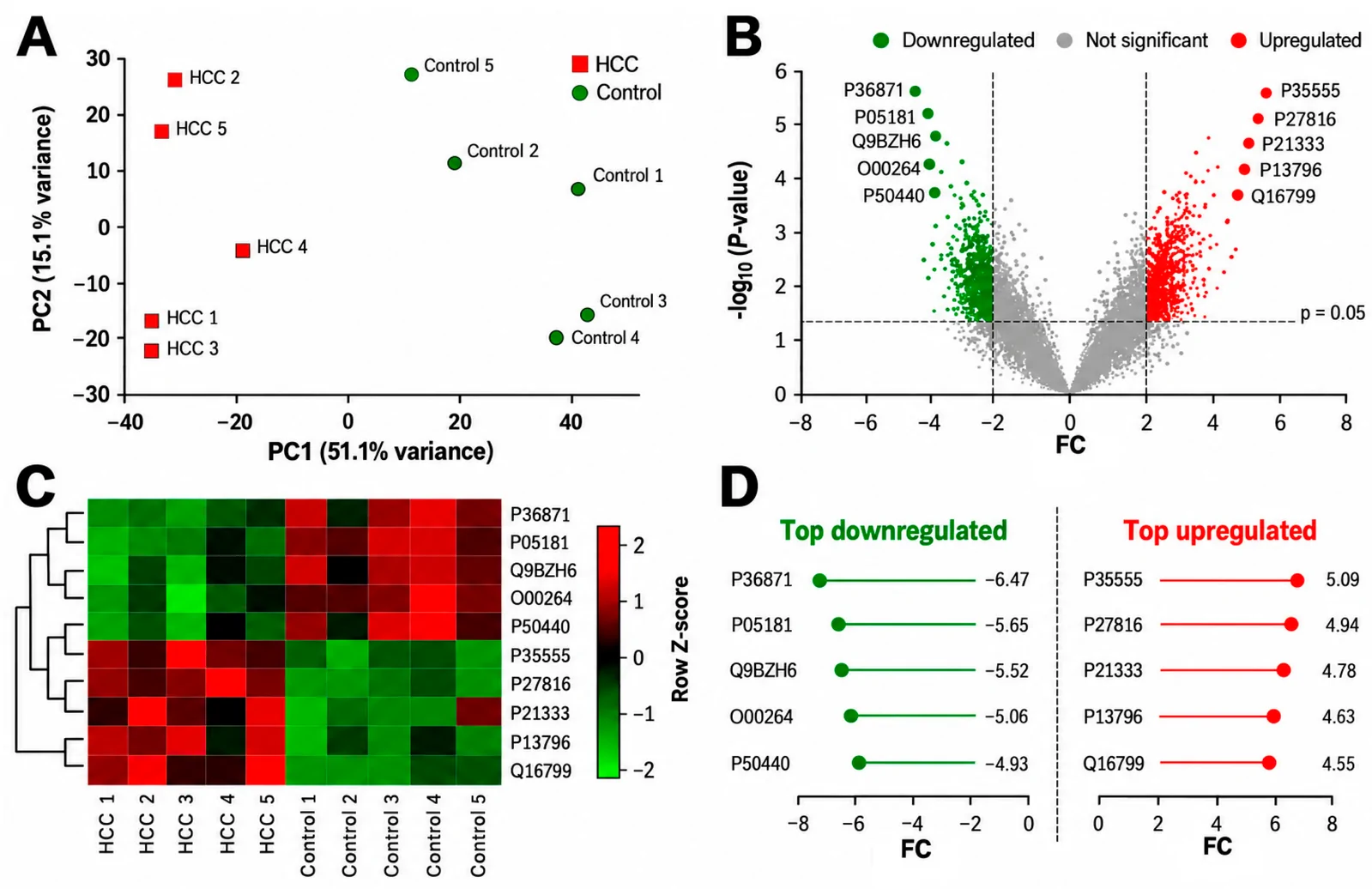
Phosphoproteome remodeling in HCC tissue. (**A**) PCA of HCC and control samples. (**B**) Volcano plot of altered proteins. (**C**) Heatmap of significant proteins. (**D**) Top 5 upregulated and downregulated proteins in HCC. FC is defined as the log_2_-transformed ratio of HCC to control values.

Following quality-control assessment and group-wise filtering, 1682 phosphopeptides were retained and used for paired statistical evaluation after missing-value imputation and normalization were performed. Among residue-type-assigned phosphorylation sites, 1767 sites were phosphoserine (pS; 92.4%), 138 were phosphothreonine (pT; 7.2%), and 8 were phosphotyrosine (pY; 0.4%), consistent with the expected predominance of serine phosphorylation in phosphoproteomic datasets. PCA of the phosphoproteome dataset showed separation between HCC and adjacent non-tumorous tissue samples, indicating that phosphorylation profiles captured disease-associated differences beyond technical variation (Figure 3A). Differential phosphopeptide analysis identified 573 phosphopeptides altered at *p* < 0.05. After applying a threshold of FDR < 0.10 and |FC| ≥ 2.0, we identified 189 phosphopeptides that were significantly altered in HCC vs. adjacent normal tissues. These included 81 increased and 108 decreased phosphopeptides (Figure 3B–D) detailed in Table S4.

The phosphopeptide volcano plot showed a broad distribution of phosphorylation changes, with both increased and decreased phosphopeptides detected in HCC tissue (Figure 3B). Compared with the whole-proteome layer, the phosphoproteome showed a larger number of significant features, suggesting that phosphorylation remodeling may represent a sensitive regulatory layer in HCC tissue. Heatmap analysis of the significant phosphopeptides demonstrated clear group-associated abundance patterns across HCC and control samples (Figure 3C). The top altered phosphopeptides included events with large fold-change values in both directions, emphasizing that HCC-associated phosphorylation remodeling involves both gain and loss of phosphorylation signals (Figure 3D). These results indicate that phosphoproteomic profiling provides additional regulatory information beyond total protein abundance.

### 2.3. Integrated Proteome-Phosphoproteome Analysis Identifies Abundance-Independent Phosphorylation Events

**Figure 4:**
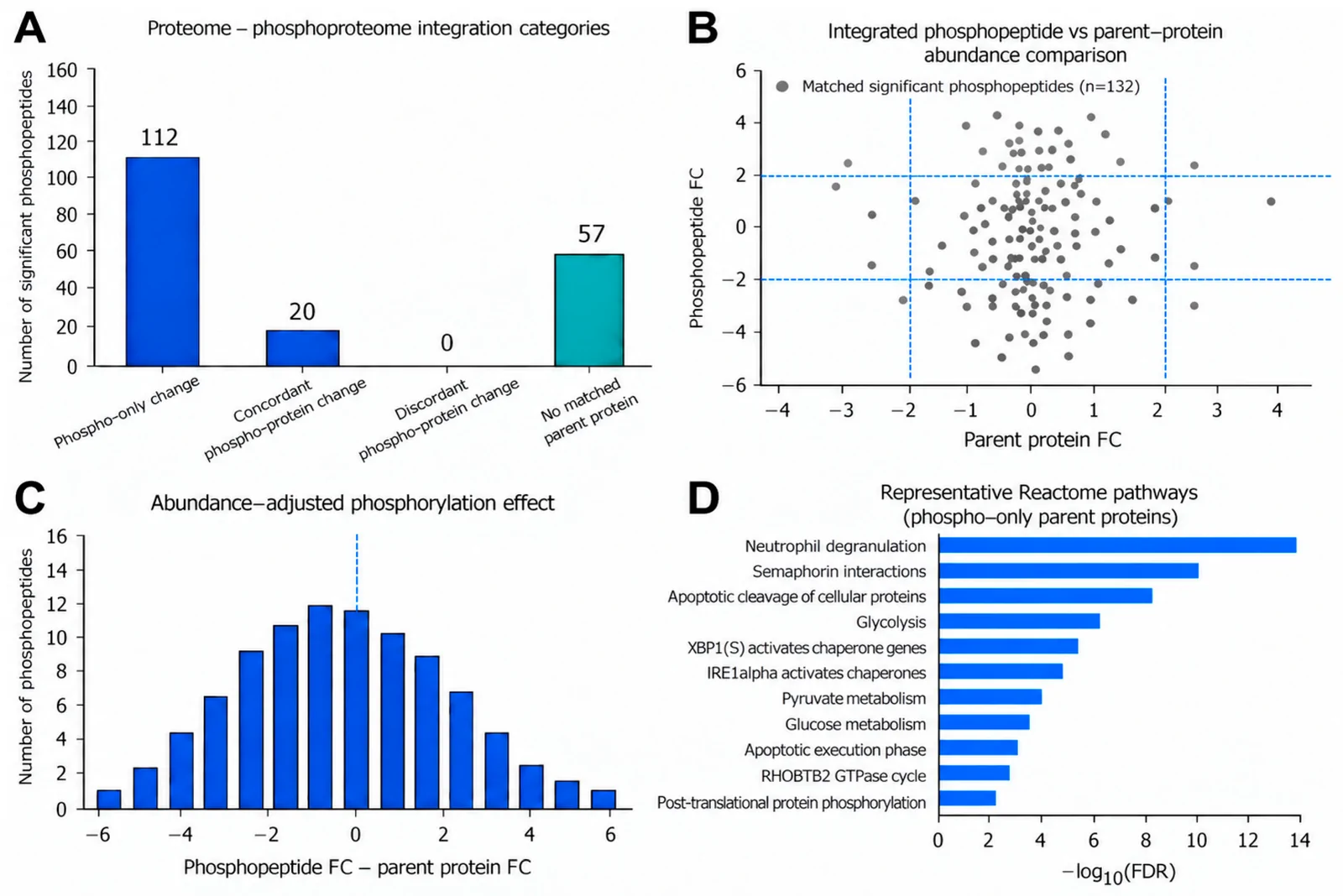
Integrated proteome–phosphoproteome analysis in HCC. (**A**) Integration category counts. (**B**) Comparison of the phosphopeptide FC and parent-protein FC values. (**C**) Abundance-adjusted phosphorylation changes. (**D**) Reactome pathways enriched among phospho-only parent proteins.

To determine whether altered phosphorylation reflected changes in parent-protein abundance or represented phosphorylation-specific regulation, significant phosphopeptides were mapped to their corresponding parent proteins quantified in the whole proteome dataset. Among the 189 significant phosphopeptides, 132 were matched to quantified parent proteins, whereas 57 did not have a matched parent-protein measurement in the whole-proteome dataset (Figure 4A). Under the applied classification criteria, 112 matched phosphopeptides were classified as phospho-only changes, indicating altered phosphorylation without a comparable change in parent-protein abundance (Table S5). While 20 phosphopeptides showed concordant phospho-plus-protein changes, no discordant phospho/protein abundance changes were detected (Figure 4A,B). These results indicate that most matched phosphorylation changes were not explained by corresponding changes in total parent-protein abundance.

The integrated scatter plot comparing phosphopeptide FC with parent-protein FC values further supported this interpretation. Most significant phosphopeptide changes occurred despite relatively limited parent-protein abundance changes, consistent with abundance-independent phosphorylation regulation (Figure 4B). Many significant phosphopeptide changes occurred despite relatively limited parent-protein abundance changes, consistent with abundance-independent phosphorylation regulation (Figure 4B). To quantify this effect, we subtracted the parent-protein FC from the phosphopeptide FC for matched events. The resulting distribution showed a broad range of abundance-adjusted phosphorylation effects, confirming that many phosphopeptide-level changes exceeded the magnitude of corresponding parent-protein changes (Figure 4C). Overall, this integrated analysis separates phosphorylation remodeling from protein-abundance remodeling and identifies candidate regulatory phosphorylation events that would not be apparent from whole-proteome analysis alone.

### 2.4. Reactome Pathway Analysis Links Phospho-Only Events to Oncogenic Signaling Pathways

To assess the biological processes represented by abundance-independent phosphorylation events, parent proteins corresponding to phospho-only phosphopeptides were subjected to Reactome pathway enrichment analysis. Of the 112 submitted parent-protein identifiers, 77 were mapped in Reactome, resulting in 524 pathway hits. In total, 12 pathways were enriched at FDR < 0.05, and 20 pathways were enriched at FDR < 0.10. Representative enriched pathways included neutrophil degranulation, semaphorin interactions, apoptotic cleavage of cellular proteins, glycolysis, XBP1(S)-mediated chaperone gene activation, IRE1α-mediated chaperone activation, pyruvate metabolism, RHO/RHOBTB GTPase-related pathways, and post-translational protein phosphorylation (Figure 4D). The full Reactome enrichment output is provided in Table S6.

These results indicate that abundance-independent phosphorylation events are associated with immune/inflammatory processes, metabolic remodeling, ER-stress/protein-folding responses, apoptosis, cytoskeletal regulation, and phosphorylation-dependent signaling. Together, the Reactome analysis supports the biological relevance of the updated phospho-only protein set and suggests that HCC-associated phosphorylation remodeling affects multiple regulatory processes beyond changes in total parent-protein abundance.

### 2.5. IPA Core Analysis Reveals Dysregulated Phosphorylation-Dependent Signaling in HCC

IPA core analysis of the 112 matched phosphoproteins identified significant enrichment of several pathways with established relevance to HCC biology (Figure S11). Among the most prominent were glycolysis I, the RHO GTPase cycle, post-translational protein phosphorylation, tight junction signaling, regulation of IGF transport and uptake by IGFBPs, and Warburg effect signaling. Additional HCC-relevant pathways included the RAF/MAP kinase cascade, the tumor microenvironment pathway, PI3K/AKT signaling, and HIF1α signaling.

Collectively, these pathways indicate coordinated alterations in metabolic regulation, growth-factor signaling, phosphorylation-dependent signaling, cytoskeletal organization, cell-junction integrity, and tumor-microenvironment interactions in the HCC phosphoproteome.

The upstream regulator network analysis provides additional evidence that the phosphoproteomic alterations observed in HCC reflect coordinated remodeling of signaling pathways rather than isolated phosphorylation changes. In particular, the *EFNA1*- and *CK2*-associated networks point to dysregulation of pathways linked to growth factor signaling, cytoskeletal organization, and tumor progression. *EFNA1* has been implicated in signaling processes that influence cell adhesion, migration, and tumor behavior, whereas *CK2* is a well-recognized serine/threonine kinase involved in proliferation, survival, and oncogenic signaling (Figure S12). Both networks connected to multiple downstream phosphoproteins, with *STMN1* emerging as a shared highlighted molecule. The presence of *STMN1* in both *EFNA1*- and *CK2*-associated networks suggests that it may serve as an important phosphorylation-sensitive node in HCC.

### 2.6. Candidate Prioritization Highlights Phospho-Only Events for Future Validation

To prioritize phospho-only events for biological interpretation and future validation, matched phosphopeptides classified as phospho-only were ranked based on the magnitude of phosphorylation change, phosphopeptide-level statistical significance, parent-protein abundance change, and the abundance-adjusted phosphorylation effect calculated as phosphopeptide FC minus parent-protein FC. The top-ranked phospho-only candidates are summarized in Table 1. These candidates showed marked phosphopeptide-level regulation in HCC without comparable significant changes in parent-protein abundance, supporting their classification as phosphorylation-specific events.

Several prioritized candidates were associated with biological processes highlighted by Reactome pathway enrichment, including immune/inflammatory activity, apoptosis, metabolic remodeling, ER-stress/protein-folding responses, RHO/RHOBTB GTPase-related regulation, and post-translational phosphorylation (Figure 4D; Table S6). These findings suggest that the prioritized phospho-only events may represent candidate regulatory phosphorylation changes associated with HCC biology and should be considered for future targeted validation.

Together, these results demonstrate that HCC tissue exhibits both protein-abundance remodeling and extensive phosphorylation-level rewiring. The integrated analysis indicates that many significant phosphorylation changes are not explained by parent-protein abundance, highlighting the value of phosphoproteomics for identifying regulatory events that may be missed by whole-proteome profiling alone.

## 3. Discussion

In this study, we performed an integrated whole-proteome and phosphoproteome analysis of paired HCC and adjacent non-tumorous tissue samples to define both protein-abundance remodeling and phosphorylation-level regulation in HCC. This paired, two-layer design allowed us to distinguish changes in total protein abundance from phosphorylation events that were not possible to decipher from only parent-protein changes. This distinction is important because phosphorylation is a rapid and reversible post-translational modification that regulates protein activity, localization, interaction networks, and signaling output, often without requiring a measurable change in total protein abundance. Consistent with previous cancer phosphoproteomics studies, our findings support the value of phosphorylation-level analysis for capturing regulatory information beyond protein expression alone [12,13,14].

Whole-proteome profiling identified a measurable protein-abundance signature separating HCC from adjacent non-tumorous tissue. After filtering, 3653 proteins were retained for paired differential analysis, and 314 proteins were identified with FDR < 0.10 and |FC| ≥ 2.0, including 79 proteins increased and 235 proteins decreased in HCC. The paired study design is a strength because it reduces inter-patient variability and focuses the analysis on tumor-associated differences within matched tissue pairs. The whole-proteome results therefore provide an important abundance reference layer for interpreting the phosphoproteome data. The phosphoproteome layer also revealed substantial disease-associated remodeling. From 1682 quantified phosphopeptides, 189 were significantly altered in HCC using the same FDR and fold-change criteria, including 81 increased and 108 decreased phosphopeptides. These findings support phosphorylation as an important regulatory layer in HCC tissue. This is biologically plausible because phosphorylation can rapidly reflect altered kinase and phosphatase activity, growth-factor signaling, metabolic regulation, cytoskeletal remodeling, and stress-response pathways. In cancer systems, quantitative phosphoproteomics has been widely used to identify aberrant signaling networks and phosphorylation events linked to tumor progression and therapeutic response [12,14,15].

The most important observation from this study was that integrated proteome–phosphoproteome analysis identified a large set of abundance-independent phosphorylation events. Among the 189 significant phosphopeptides, 132 could be mapped to quantified parent proteins, allowing direct comparison between phosphopeptide changes and corresponding parent-protein abundance changes. Of these matched events, 112 were classified as phospho-only changes, whereas 20 showed concordant phosphoprotein abundance changes. The phospho-only events showed altered phosphopeptide abundance without comparable changes in total parent-protein abundance, indicating that these changes were more consistent with phosphorylation-specific regulation than with altered protein expression alone. This distinction is critical because phosphopeptide changes can be misinterpreted if parent-protein abundance is not considered. By comparing phosphopeptide and parent-protein FC values and calculating the phospho-minus-protein effect, we prioritized phosphorylation events that may reflect HCC-associated regulatory remodeling beyond changes in protein quantity.

This abundance-adjusted interpretation is a major strength of the study. A phosphopeptide increase in HCC could reflect either increased phosphorylation stoichiometry or increased abundance of the parent protein. Conversely, a phosphopeptide that changes while the parent protein remains relatively stable provides stronger evidence for phosphorylation-level regulation. Integrated proteomic and phosphoproteomic approaches improve pathway interpretation by separating abundance changes from post-translational regulation, and large-scale tumor proteogenomic studies have shown that phosphorylation can reveal regulatory information not captured by protein abundance alone [3,15,16]. In this context, the phospho-only events identified in our study likely represent candidate regulatory phosphorylation changes associated with HCC tissue remodeling.

Previous HCC proteogenomic and phosphoproteomic studies have shown that liver tumors exhibit broad proteomic and phosphorylation-level alterations associated with tumor biology, molecular subtypes, and cancer-related pathways [3,16,17]. Our study is complementary to these reports but differs in its direct paired integration of whole-proteome and phosphoproteome measurements to distinguish phosphorylation changes from parent-protein abundance changes. This approach identified 112 phospho-only events whose regulation was not explained by comparable changes in total parent-protein abundance. Therefore, the main novel contribution of this study is the identification and prioritization of abundance-independent phosphorylation events in paired HCC tissue.

Reactome pathway analysis further supported the biological relevance of the phospho-only events. Parent proteins corresponding to phospho-only phosphopeptides were associated with immune/inflammatory activity, metabolic remodeling, ER-stress/protein-folding responses, apoptosis, RHO/RHOBTB GTPase-related regulation, and post-translational phosphorylation (Figure 4D; Table S6). These pathway themes are relevant to HCC biology because tumor progression is accompanied by changes in inflammatory signaling, metabolic adaptation, cellular stress responses, apoptosis regulation, cytoskeletal organization, and phosphorylation-dependent regulatory networks [3,16,18]. Therefore, the pathway enrichment results suggest that the phospho-only events identified here are not random phosphorylation changes but are connected to biological processes relevant to HCC-associated tissue remodeling.

The Reactome-based interpretation also strengthens the integrated analysis because it links individual phosphorylation events to broader pathway-level biology. Reactome is a curated pathway knowledge base that supports systematic interpretation of molecular datasets, and Reactome-based enrichment tools are commonly used to identify overrepresented biological pathways from omics-derived protein lists [19,20,21]. In our study, Reactome analysis indicated that abundance-independent phosphorylation events were associated with multiple regulatory processes rather than nonspecific protein changes alone. This finding supports the conclusion that HCC tissue undergoes phosphorylation-level remodeling across immune, metabolic, stress-response, apoptotic, cytoskeletal, and phosphorylation-related pathways, even when corresponding parent-protein abundance changes are limited.

The upstream regulator analysis further supports altered kinase- and growth factor-mediated signaling in HCC. Several HCC-relevant regulators were identified, including the *CK2* complex/*CSNK2A1*, *EFNA1*, *CDK5*, and *PAK2*, which are associated with cellular proliferation, survival, cytoskeletal organization, and migratory signaling. The prominence of *CK2*-related regulators is particularly consistent with widespread disruption of phosphorylation-dependent signaling, while *CDK5* and *PAK2* further suggest alterations in kinase networks involved in cellular growth and structural remodeling. Notably, STMN1 was common to both networks, highlighting its potential role as a convergent downstream effector. Because STMN1 is involved in microtubule dynamics, cell-cycle progression, and tumor cell motility, its altered phosphorylation may contribute to HCC progression and warrants further investigation. Given the limited number of paired samples, these observations are best considered hypothesis-generating and will require validation in larger cohorts and with pathway-specific functional assays.

The prioritized phospho-only candidate events summarized in Table 1 provide a focused set of phosphorylation changes for future validation. Candidates were ranked based on phosphopeptide FC, statistical significance, parent-protein FC, and the phospho-minus-protein effect size. This prioritization strategy is important because the most biologically informative events are not necessarily those with the largest phosphopeptide FC alone but those showing strong phosphorylation regulation relative to parent-protein abundance. Several high-priority candidates showed large abundance-adjusted phosphorylation changes, supporting their potential value as candidate phosphorylation-specific markers of HCC-associated regulatory remodeling. These events should be considered for targeted validation using PRM- or MRM-based phosphoproteomics and, where suitable antibodies exist, orthogonal validation using phosphosite-specific immunoassays.

This study has several strengths. The paired HCC and adjacent non-tumorous design reduced inter-individual variability and improved detection of tumor-associated molecular differences. In addition, parallel whole-proteome and phosphoproteome profiling of the same sample set enabled interpretation of phosphopeptide changes in the context of parent protein abundance. This integration reduced the risk of overinterpreting phosphorylation changes that may simply reflect protein-abundance differences and helped prioritize candidate phospho-only events for follow-up validation.

The main limitation of this study is the small sample size; therefore, the findings should be considered discovery-level until validated in an independent cohort. Although paired statistical testing improves interpretability, larger studies will be needed to assess inter-tumor heterogeneity and detect more subtle changes. In addition, data-dependent acquisition may undersample low-abundance proteins and phosphopeptides, and some significant phosphopeptides may not be matched to quantified parent proteins due to proteome coverage limitations. Because phosphorylation stoichiometry or site occupancy was not directly measured, the term “abundance-independent phosphorylation” is used to operationally describe phosphopeptide changes that were not explained by comparable matched parent-protein abundance changes, rather than as direct evidence of altered phosphorylation stoichiometry or site occupancy. The prioritized phospho-only events should be validated in future studies using targeted phosphoproteomics approaches such as PRM/MRM and, where suitable phosphosite-specific antibodies are available, by Western blotting or phosphosite-specific immunoassays. Finally, phosphoproteomics does not directly measure kinase activity, so pathway- and kinase-related interpretations should be viewed as hypothesis-generating and require validation by kinase-substrate analysis or functional assays.

## 4. Methods and Materials

### 4.1. Tissue Samples and Protein Extraction

Adult patients recruited to participate in this study at MedStar Georgetown University Hospital signed informed consent to a protocol approved by the Georgetown University Institutional Review Board (IRB Approval # 2005-206) on 15 January 2026. Following the participant’s informed consent signature and enrollment, liver tissues were collected at the time of the surgical procedure and stored in liquid nitrogen until the day of processing. HCC cases were diagnosed based on diagnostic imaging criteria or histology. In this study, fresh-frozen tissues and matched adjacent non-tumorous liver tissues from five subjects were analyzed. Tissue specimens were handled under cold conditions throughout processing. Each sample was first pulverized in liquid nitrogen and then lysed in RIPA buffer (Thermo Fisher Scientific, Waltham, MA, USA, Cat. No. 89900), which was supplemented immediately before use with Halt™ protease and phosphatase inhibitor cocktails, EDTA-free (Thermo Fisher Scientific, Waltham, MA, USA, USA Cat. No. PI78441), to minimize protein degradation and ex vivo dephosphorylation during lysis. Total protein concentration was determined using the Micro BCA Protein Assay Kit (Thermo Fisher Scientific, Waltham, MA, USA, Cat. No. 23235).

Patient-level demographic, clinical, radiologic, laboratory, and histopathologic characteristics are summarized in Supplementary Table S7. The five patients were men aged 47–68 years, and all had underlying cirrhosis associated with hepatitis C virus infection, alcohol-associated liver disease, or nonalcoholic steatohepatitis. Available viral hepatitis serology, AFP, MELD, AST, and ALT values are also reported. Because of the small and clinically heterogeneous cohort and the limited availability of standardized clinical annotations, the proteomic and phosphoproteomic findings should be interpreted as exploratory and hypothesis-generating.

### 4.2. Sample Preparation

For whole-proteomic analysis, 50 μg of total protein from each sample was processed using a filter-aided sample preparation (FASP) workflow following our previously reported paper [22]. Briefly, protein lysates were diluted in 7 M urea and 100 mM ammonium bicarbonate and loaded onto a 30 kDa molecular weight cutoff filter (Amicon Ultra, MilliporeSigma, Burlington, MA, USA, Cat. No. UFC803008). Reduction was performed with 100 μL of 20 mM dithiothreitol (DTT) for 60 min at room temperature, followed by centrifugation at 14,000× *g* for 10 min. Alkylation was then carried out with 100 μL of 30 mM iodoacetamide (IAA) in the dark at room temperature. After alkylation, the samples were washed twice with 50 mM ammonium bicarbonate and once with 10 mM ammonium bicarbonate. The final solution was diluted with 50 μL of ultrapure water, and proteins were digested using Trypsin/Lys-C Mix (Thermo Fisher Scientific, Waltham, MA, USA, Cat. No. A40009) at an enzyme-to-protein ratio of 1:50. After digestion, peptides were acidified and stored at 20 °C until desalting. After digestion, peptides were desalted using C18 solid-phase extraction, dried, reconstituted in 3% ACN/0.1% FA, quantified by NanoDrop (Thermo Fisher Scientific), and 1 μg was injected for each nanoLC-MS/MS run. Each sample was analyzed in three technical replicate injections. A Pierce HeLa Protein Digest Standard (Thermo Fisher Scientific, Cat. No. 88328) was analyzed before, during, and after the patient samples to monitor chromatographic stability and instrument performance.

For phosphoproteomic analysis, 150 μg of total protein from each tissue sample was processed using the same FASP-based digestion workflow described above. Following digestion, phosphopeptides were enriched using the High-Select Fe-NTA Phosphopeptide Enrichment Kit (Thermo Fisher Scientific, Waltham, MA, USA, Cat. No. A32992), which uses ferric ion–phosphate interactions and provides a reproducible workflow compatible with limited tissue-derived peptide input using the manufacturer’s recommended protocol. Enriched phosphopeptides were dried, reconstituted in 3% ACN/0.1% FA, quantified by NanoDrop (Thermo Fisher Scientific, Waltham, MA, USA), and 1 μg was injected for each nanoLC-MS/MS run.

### 4.3. nanoLC-MS/MS Analysis

Peptides were analyzed on an UltiMate 3000 UHPLC system coupled to a Q Exactive Orbitrap mass spectrometer equipped with a Nanospray Flex ion source (Thermo Fisher Scientific, San Jose, CA, USA). Peptides were first trapped on a C18 Acclaim PepMap trap column (75 μm × 20 mm, 100 Å) and then separated on a C18 Acclaim PepMap RSLC analytical column (75 μm × 250 mm, 100 Å) maintained at 35 °C. Mobile phase A consisted of 0.1% FA in water, and mobile phase B consisted of 80% ACN containing 0.1% FA. A 145 min multistep gradient was used. Mobile phase B was held at 4% for 5 min at 300 nL/min, increased to 35% over 120 min at 220 nL/min, increased to 95% over 5 min at 250 nL/min, and then returned to initial conditions for re-equilibration. The mass spectrometer was operated in data-dependent acquisition (DDA) mode. A spray voltage of 2.2 kV was applied. Full MS scans were acquired over an m/z range of 370–1850 in the Orbitrap at a resolution of 70,000. For MS/MS acquisition, the 10 most intense precursor ions were selected for higher-energy collisional dissociation (HCD) using a normalized collision energy (NCE) of 27.5, and fragment ions were acquired at a resolution of 17,500. These match the earlier Q Exactive method. The same chromatographic method and DDA settings were used for whole-proteome, phosphoproteome, and QC analyses to match the earlier 145 min gradient method.

### 4.4. Protein and Phosphoprotein Identification

Raw data were processed in Proteome Discoverer 3.0 (Thermo Fisher Scientific, Waltham, MA, USA) using a label-free quantification (LFQ) workflow with the Sequest search engine. MS/MS spectra were searched against the UniProt human proteome FASTA database supplemented with a common contaminant sequence list. Trypsin was specified as the digestion enzyme, with a precursor mass tolerance of 10 ppm and a fragment mass tolerance of 0.02 Da. Carbamidomethylation of cysteine was included as a fixed modification. For phosphoproteomics data, phosphorylation of serine, threonine, and tyrosine (+79.966 Da) was included as a variable modification for phosphopeptide identification. Phosphosite localization confidence was evaluated in Proteome Discoverer, and phosphopeptides were further filtered to retain only sites with localization probability > 0.98; phosphopeptides with lower probability or ambiguous site assignment were excluded from downstream site-level analysis. The processing workflow included mass recalibration, feature detection, peptide-spectrum matching, and confidence filtering. Protein identifications in the global proteomics dataset were assigned from matched peptide-spectrum assignments, whereas phosphoproteome identifications were derived from the corresponding phosphorylated peptide-spectrum matches detected in the enriched phosphopeptide runs. High-confidence identifications were retained using an FDR threshold of 1%.

### 4.5. Data Processing and Analysis

Protein- and phosphopeptide-level abundance data tables exported from Proteome Discoverer were processed separately for whole-proteome and phosphoproteome analyses. Technical replicate performance was first evaluated using quality-control metrics, including feature detection counts, abundance distributions, within-sample replicate correlations, correlation heatmaps, and coefficient-of-variation analysis. Following quality-control assessment, technical replicate measurements were collapsed to the biological sample level by calculating arithmetic mean abundance for each protein or phosphopeptide. Features were then filtered using a group-wise 70% presence criterion, retaining proteins and phosphopeptides detected in at least 70% of samples within either the HCC or non-tumorous group for subsequent analysis. Following data filtering, 636 of 36,530 proteome values (1.7%) and 462 of 16,820 phosphoproteome values (2.7%) were missing. Given the relatively low proportion of missing values, feature-wise K-nearest neighbors (KNN) imputation with K = 5 was performed, in which missing values were estimated from the five most similar features across samples. The imputed abundance values were then log_2_-transformed and quantile-normalized prior to statistical testing.

Differential abundance analysis was performed using a paired two-sided Student’s *t*-test to account for matched HCC and non-tumorous tissue pairs. Multiple-testing correction was performed using the Benjamini–Hochberg false-discovery-rate method. Proteins and phosphopeptides were considered significantly altered at an FDR < 0.10 and |FC| ≥ 2.0, where FC denotes the log_2_-transformed ratio of HCC to control abundance values.

For integrated analysis, significant phosphopeptides were mapped to quantified parent proteins using protein accession identifiers, and phosphopeptide FC values were compared with corresponding parent-protein FC values to identify abundance-independent phosphorylation events. Differentially phosphorylated proteins identified from the phosphoproteomic analysis were uploaded into Reactome for pathway analysis. In addition, Ingenuity Pathway Analysis (IPA, QIAGEN, Hilden, Germany) core analysis was performed. Molecules were mapped to the IPA Knowledge Base to interpret phosphorylation-associated signaling changes in HCC tissues compared with adjacent non-tumorous tissues. Statistical enrichment was determined using Fisher’s exact test, and predicted activation states were evaluated using IPA activation z-scores.

## 5. Conclusions

In conclusion, this study shows that paired HCC and adjacent non-tumorous liver tissues exhibit both protein-abundance remodeling and phosphorylation-level rewiring. Whole-proteome profiling identified 314 significantly altered proteins, whereas phosphoproteome profiling identified 189 significantly altered phosphopeptides. Integrated analysis identified 112 phospho-only events whose regulation was not explained by comparable changes in parent-protein abundance. Reactome analysis linked these abundance-independent phosphorylation events to immune/inflammatory activity, metabolic remodeling, ER-stress/protein-folding responses, apoptosis, RHO/RHOBTB GTPase-related regulation, and post-translational phosphorylation. Overall, these findings support integrated proteome–phosphoproteome profiling as a useful strategy for distinguishing protein-abundance changes from phosphorylation-specific regulation and provide candidate phosphorylation events for future validation in HCC.

## Figures and Tables

**Table 1:** Prioritized phospho-only candidate events identified by integrated proteome–phosphoproteome analysis.

Rank	Gene ID	Phosphopeptide Sequence	Phosphosite/Modification	Phospho-Peptide FC	Protein FC	Phospho-Protein FC	Phospho-Peptide FDR	Parent Protein FDR	Priority Score *
1	*TOM1*	AADRLPNLSSPSAEGPPGPPSGPAPR	Phospho [S462]	4.06	−1.06	5.12	0.061	0.444	11.60
2	*RRBP1*	KAEGSPSEGK	Phospho [S573]	−4.81	−0.71	−4.11	0.061	0.172	11.34
3	*PGRMC1*	GDQPAASGDSDDDEPPPLPR	Phospho [S54]	−5.06	−1.27	−3.79	0.061	0.082	11.27
4	*MAP4*	SKVGSTENIK	Phospho [S941]	4.94	1.06	3.88	0.061	0.087	11.24
5	*TGOLN2*	SSAEAQTPEDTPNK	Phospho [S70]	−4.12	0.77	−4.89	0.083	0.217	11.18
6	*RTN1*	GSISEDELITAIK	Phospho [S350]	4.55	0.45	4.11	0.061	0.792	11.08
7	*GPI*	SNTPILVDGK	Phospho [T109]	−4.61	−0.75	−3.86	0.061	0.209	10.89
8	*SPAG9*	SASQSSLDKLDQELK	Phospho [S730]	−3.81	0.83	−4.64	0.061	0.484	10.87
9	*TJP2*	AASSDQLR	Phospho [S978]	−4.13	0.22	−4.35	0.065	0.770	10.87
10	*PTMS*	SVEAAAELSAK	Phospho [S13]	−4.49	−0.70	−3.79	0.061	0.452	10.71
11	*GYG2*	VKELSPEEER	Phospho [S468]	−4.31	−0.36	−3.95	0.065	0.906	10.63
12	*GPI*	TFTTQETITNAETAK	Phospho [T214]	−4.46	−0.75	−3.71	0.061	0.209	10.60
13	*CDS2*	VDGETASDSESR	Phospho [S33]	−3.47	1.39	−4.87	0.079	0.594	10.55
14	*TGOLN2*	SGAEEQGPIDGPSK	Phospho [S224]	−3.65	0.77	−4.42	0.061	0.217	10.49
15	*LCP1*	MARGSVSDEEMMELR	Phospho [S7]	4.63	1.01	3.62	0.079	0.247	10.45

* Priority score is calculated as |phosphopeptide FC| + |phosphopeptide FC − parent-protein FC| + 2 × [−log10(phosphopeptide FDR)].

## Data Availability

The mass spectrometry proteomics data generated in this study have been deposited in the ProteomeXchange Consortium via the PRIDE partner repository with the dataset identifier [PXD081942].

## Supplementary Materials

The following supporting information can be downloaded at https://www.mdpi.com/article/10.3390/ijms27208977/s1.
