# Supplementary material for: Integrated Proteome and Phosphoproteome Profiling Reveals Abundance-Independent Phosphorylation Remodeling in Hepatocellular Carcinoma
Source: Int J Mol Sci. 2026 Oct 10;27(20):8977. doi: 10.3390/ijms27208977 (PMC13705115; doi:10.3390/ijms27208977)
Supplement: Supplementary file 1 [file ijms-27-08977-s001.zip › ijms-4440922-supplementary.pdf]

Integrated Proteome and Phosphoproteome Profiling Reveals Abundance-Independent Phosphorylation Remodeling in Hepatocellular Carcinoma

Shafaq Saleem[1], Muhammad Salman Sajid[1], Rency S. Varghese[1], Lin Liu[1], Vinona Muralidaran[2,3], Alexander Kroemer[2], Habtom W. Ressom[1*]

[1]Department of Oncology, Lombardi Comprehensive Cancer Center, Georgetown University Medical Center, Washington, DC 20057, USA

2MedStar Georgetown Transplant Institute, MedStar Georgetown University Hospital, Washington, DC 20057, USA

3The Center for Translational Transplant Medicine, Georgetown University Medical Center, Washington, DC 20057, USA

***Corresponding Author**
Prof. Habtom W. Ressom
Department of Oncology
Lombardi Comprehensive Cancer Center
Georgetown University Medical Center
Washington, DC 20057, USA
Email: hwr@georgetown.edu

Table of Content

Figure S1: Within-sample technical replicate CVs showing quantitative reproducibility across whole-proteome biological samples. **3**

Figure S2: Whole-proteome normalization metrics comparing median range, PCA separation, and median CV across preprocessing strategies. **3**

Figure S3: Whole-proteome protein detection counts across technical LC–MS/MS runs. **4**

Figure S4: Whole-proteome technical-run correlation heatmap showing Pearson correlation-based reproducibility across replicate LC–MS/MS runs. **4**

Figure S5: Whole-proteome normalization comparison showing improved sample abundance alignment after normalization. **5**

Figure S6: Within-sample technical replicate CVs showing reproducible phosphopeptide quantification across biological samples. **5**

Figure S7: Phosphoproteome normalization metrics comparing median range, IQR range, median CV, and PCA separation across preprocessing strategies. **6**

Figure S8: Phosphopeptide detection counts across technical LC–MS/MS runs. **6**

Figure S9: Phosphoproteomics technical-run correlation heatmap showing Pearson correlation-based reproducibility across replicate LC–MS/MS runs. **7**

Figure S10: Phosphoproteome normalization comparison showing phosphopeptide abundance distribution alignment across preprocessing strategies. **7**

Figure S11: HCC-relevant canonical pathways identified by Ingenuity Pathway Analysis based on their biological relevance to hepatocellular carcinoma. Bars represent pathway enrichment significance expressed as −log10(p-value). Higher values indicate stronger enrichment in the HCC phosphoproteomic dataset. **8**

Figure S12: Upstream regulator networks associated with altered phosphorylation in HCC. (A) EFNA1-centered upstream regulator network showing downstream phosphoproteins with altered phosphorylation in HCC. (B) CK2-centered upstream regulator network showing associated downstream phosphoproteins with altered phosphorylation in HCC. **9**

Table S1: Proteins identified from proteomics analysis. **XLS**

Table S2: Significant proteins. **XLS**

Table S3: Phosphopeptides identified. **XLS**

Table S4: Significant phosphopeptides. **XLS**

Table S5: Phospho-only change proteins. **XLS**

Table S6: Reactome enrichment output. **XLS**

Table S7: Patient-level demographic, clinical, laboratory, treatment, and histopathologic characteristics of the five paired liver tissue cases included in the proteomic and phosphoproteomic analyses. **XLS**

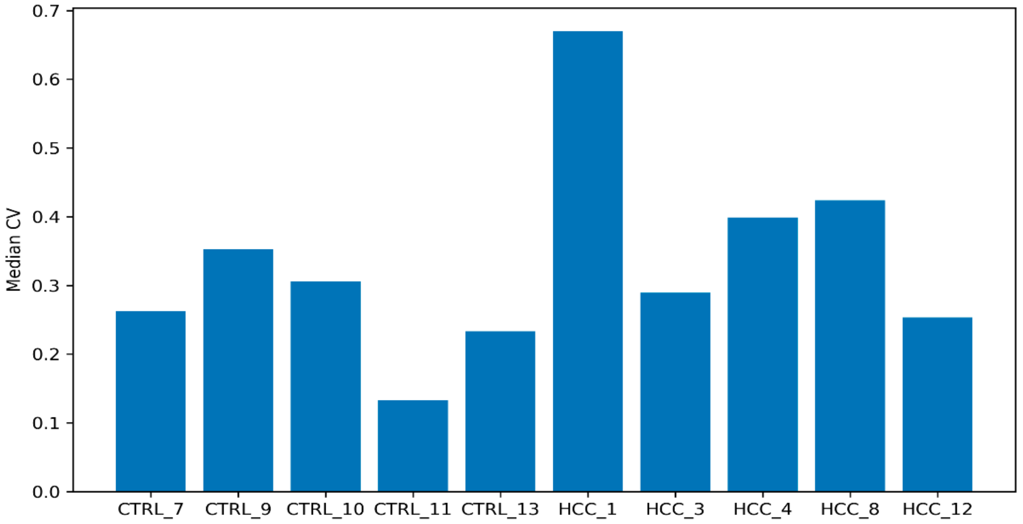

Figure S1: Within-sample technical replicate CVs showing quantitative reproducibility across whole-proteome biological samples.

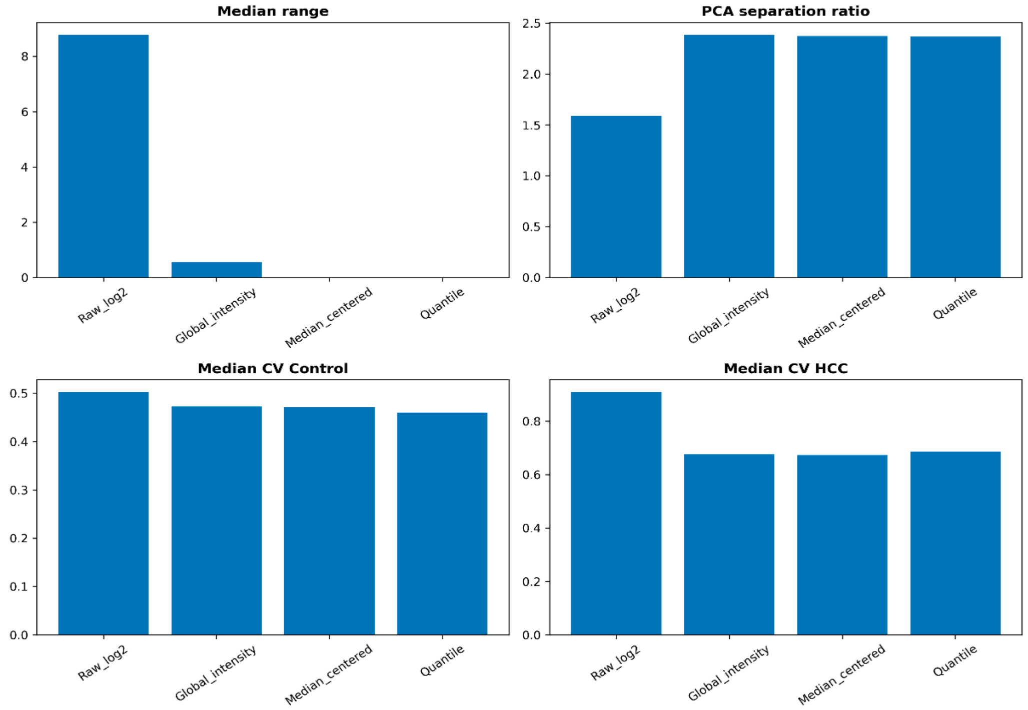

Figure S2: Whole-proteome normalization metrics comparing median range, PCA separation, and median CV across preprocessing strategies.

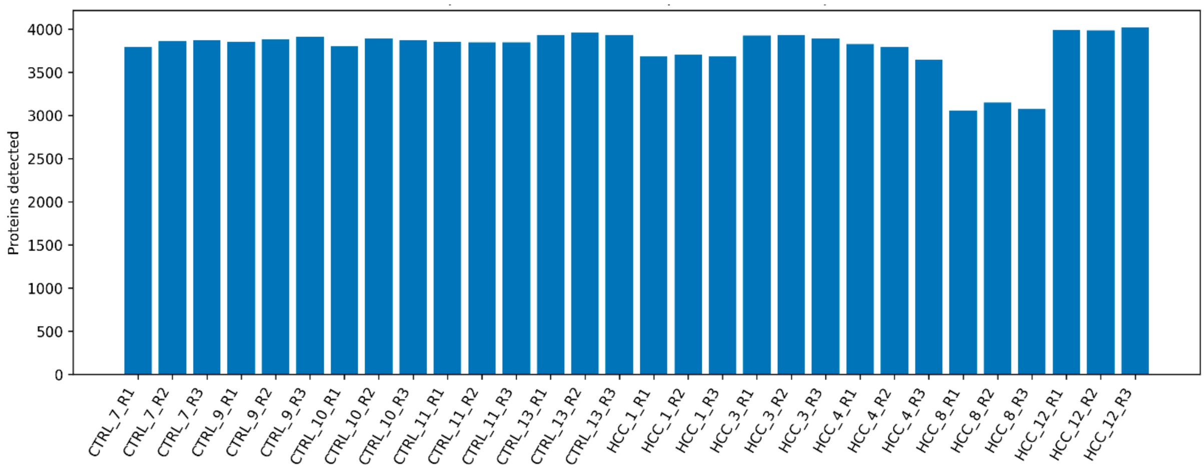

Figure S3: Whole-proteome protein detection counts across technical LC–MS/MS runs.

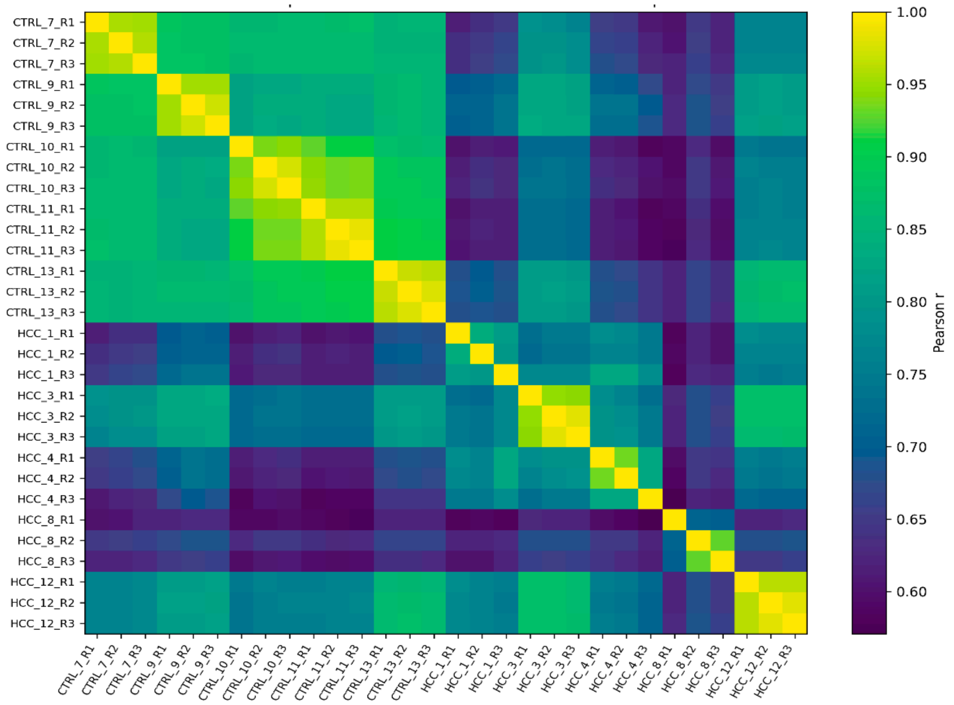

Figure S4: Whole-proteome technical-run correlation heatmap showing Pearson correlation-based reproducibility across replicate LC–MS/MS runs.

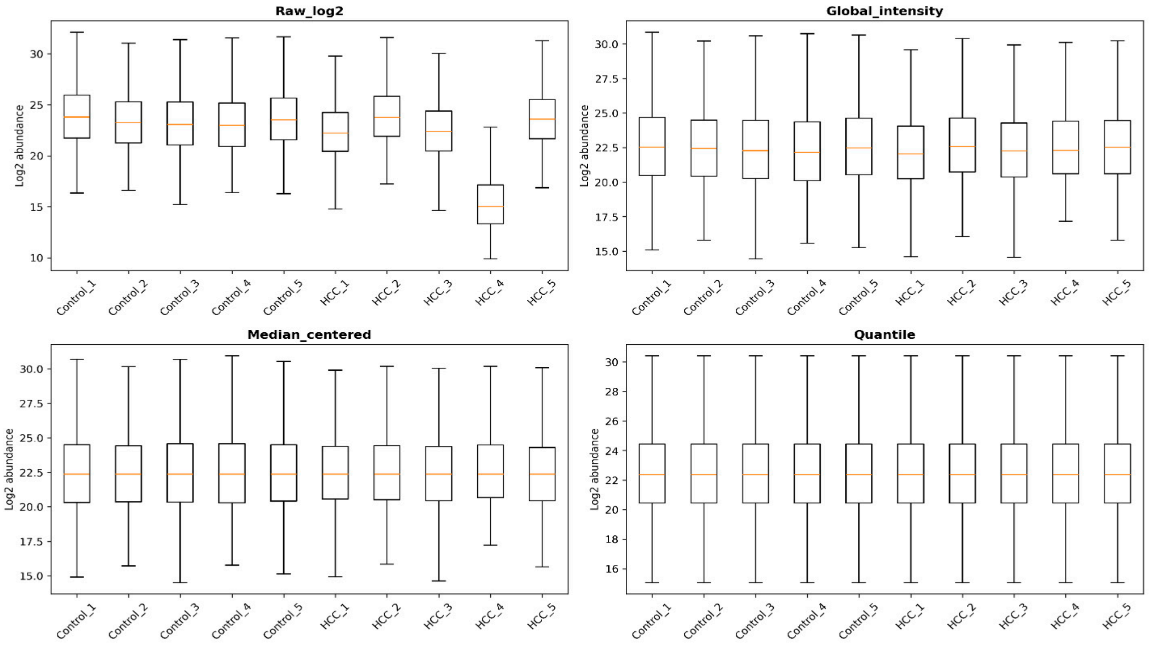

Figure S5: Whole-proteome normalization comparison showing improved sample abundance alignment after normalization.

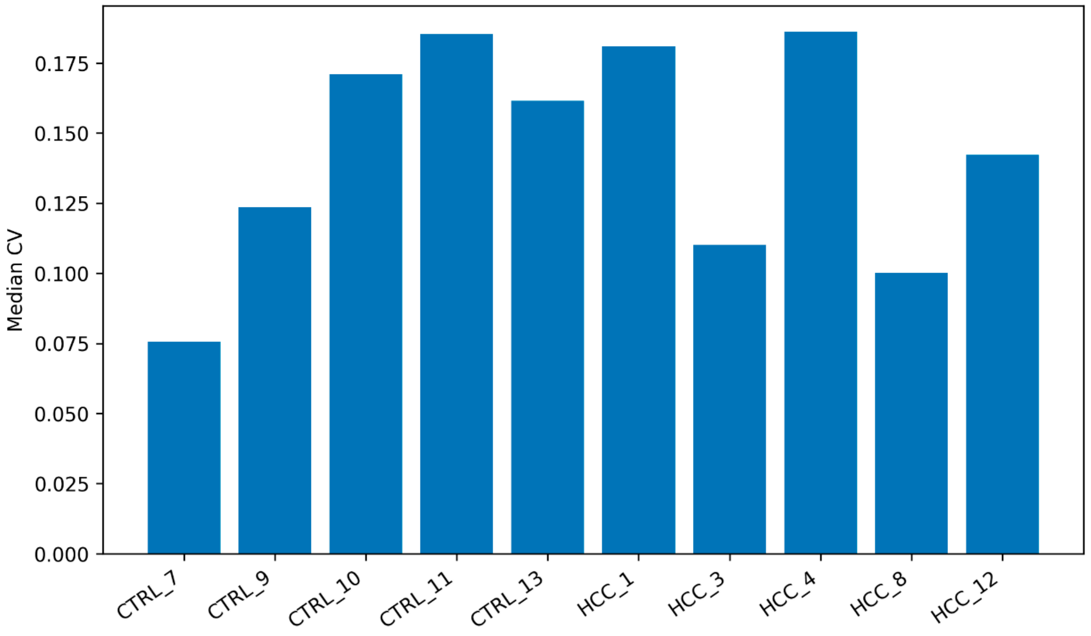

Figure S6: Within-sample technical replicate CVs showing reproducible phosphopeptide quantification across biological samples.

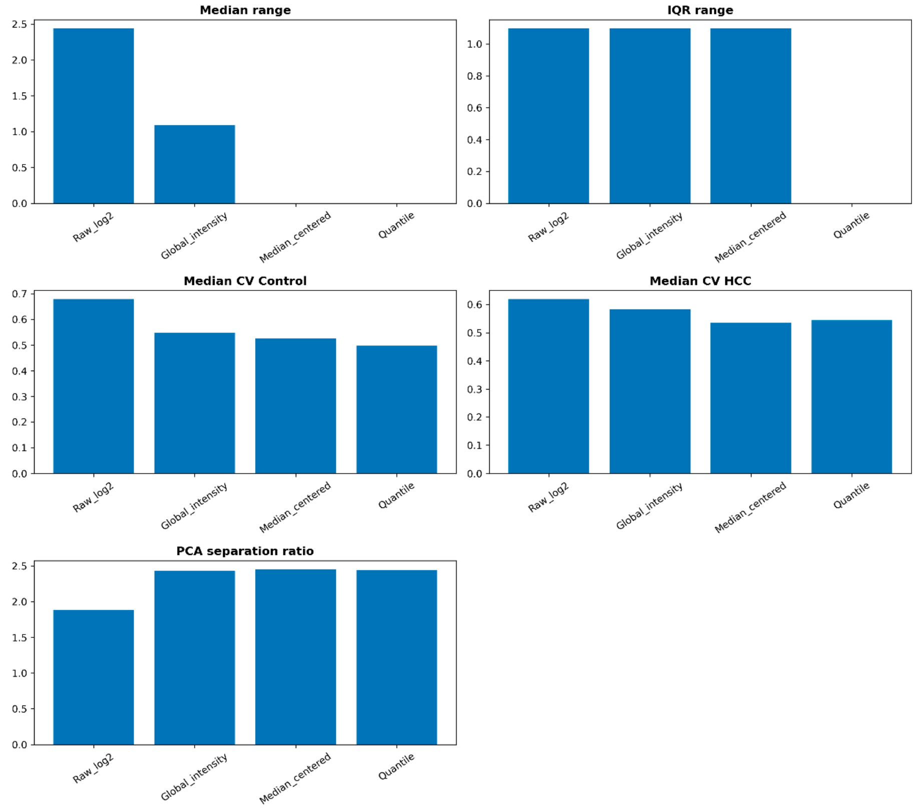

Figure S7: Phosphoproteome normalization metrics comparing median range, IQR range, median CV, and PCA separation across preprocessing strategies.

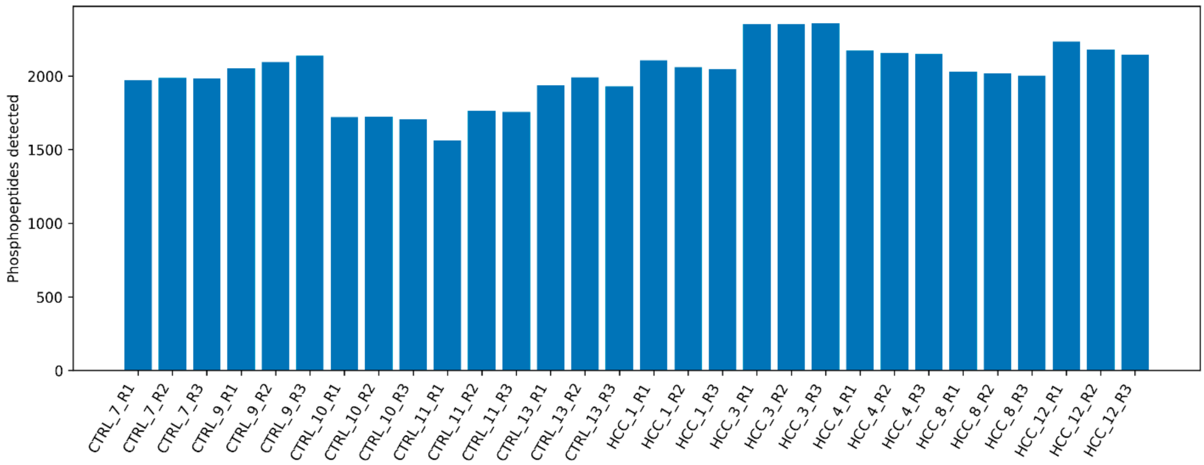

Figure S8: Phosphopeptide detection counts across technical LC–MS/MS runs.

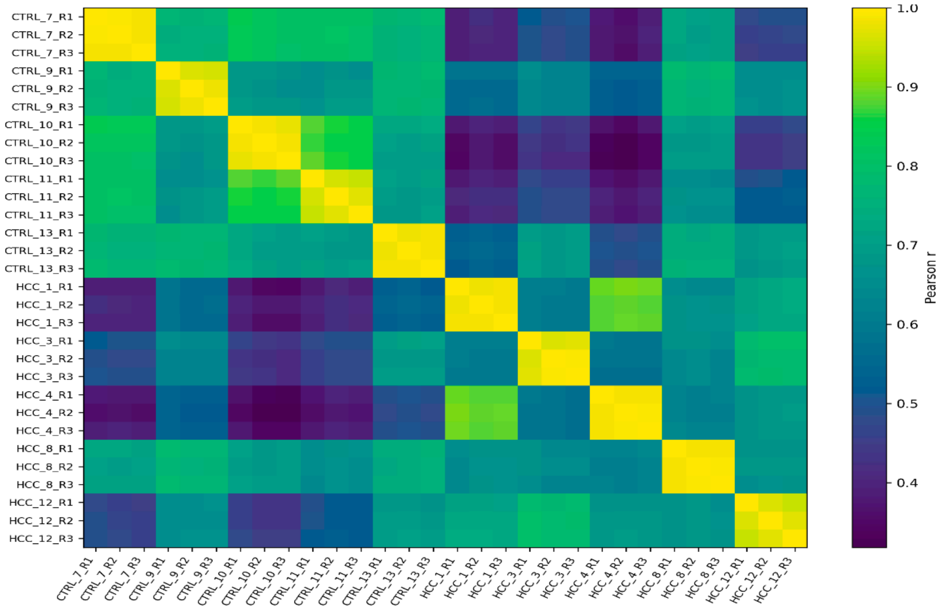

Figure S9: Phosphoproteomics technical-run correlation heatmap showing Pearson correlation-based reproducibility across replicate LC–MS/MS runs.

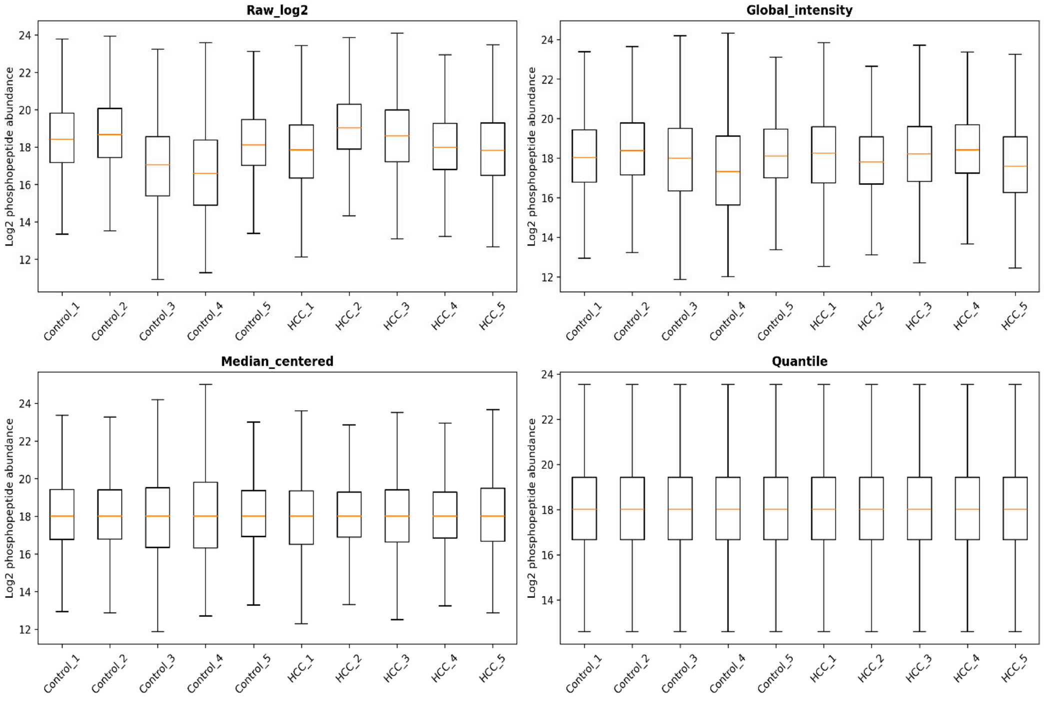

Figure S10: Phosphoproteome normalization comparison showing phosphopeptide abundance distribution alignment across preprocessing strategies.

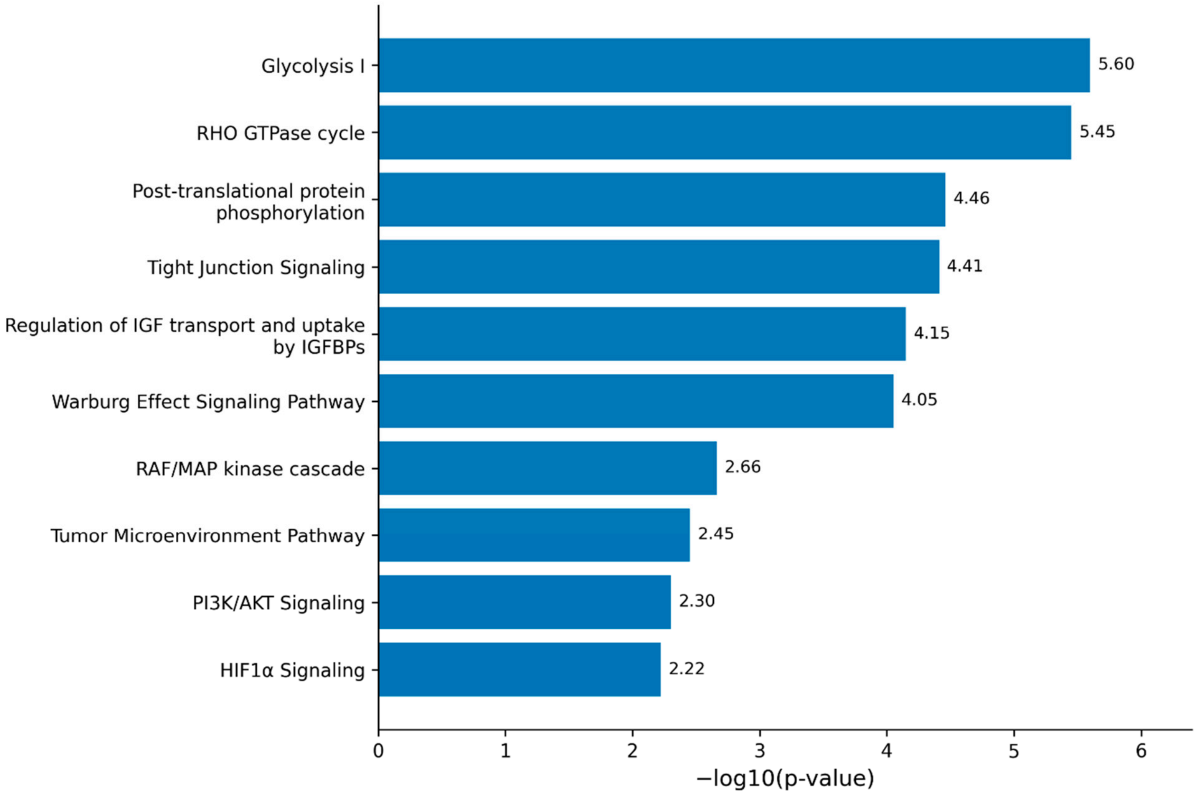

Figure S11: HCC-relevant canonical pathways identified by Ingenuity Pathway Analysis based on their biological relevance to hepatocellular carcinoma. Bars represent pathway enrichment significance expressed as −log10(p-value). Higher values indicate stronger enrichment in the HCC phosphoproteomic dataset.

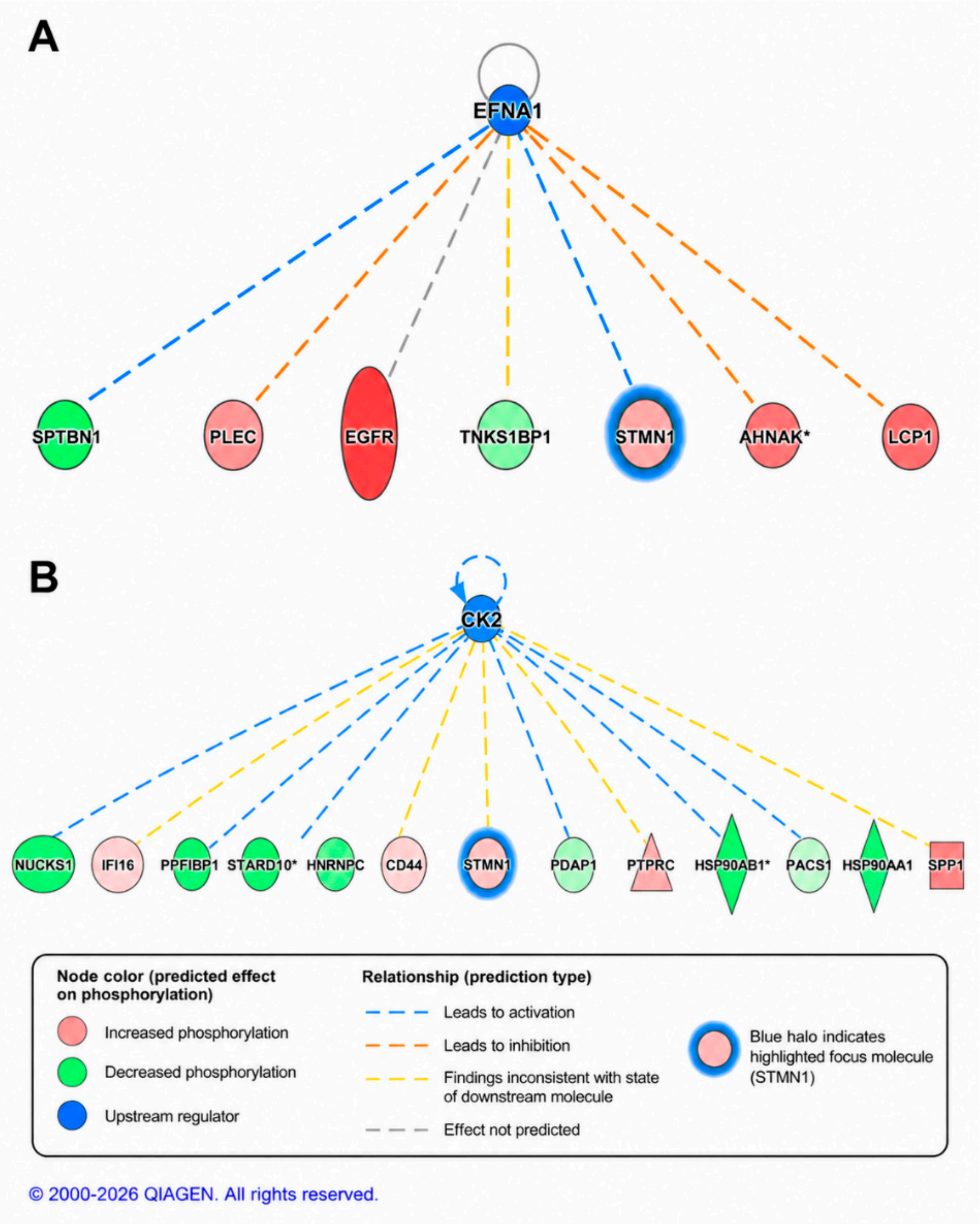

Figure S12: Upstream regulator networks associated with altered phosphorylation in HCC. (A) EFNA1-centered upstream regulator network showing downstream phosphoproteins with altered phosphorylation in HCC. (B) CK2-centered upstream regulator network showing associated downstream phosphoproteins with altered phosphorylation in HCC.
